# Prevalence of Potentially Inappropriate Medication use in older drivers

**DOI:** 10.1186/s12877-019-1287-8

**Published:** 2019-10-10

**Authors:** Guohua Li, Howard F. Andrews, Stanford Chihuri, Barbara H. Lang, Cheng Shiun Leu, David P. Merle, Abigail Gordon, Thelma J. Mielenz, David Strogatz, David W. Eby, Marian E. Betz, Carolyn DiGuiseppi, Vanya C. Jones, Lisa J. Molnar, Linda L. Hill, The LongROAD Research Team, David LeBlanc, Lindsay Ryan

**Affiliations:** 1 Department of Anesthesiology, Columbia University College of Physicians and Surgeons, New York, NY USA; 2 Department of Epidemiology, Columbia University Mailman School of Public Health, New York, NY USA; 3 Center for Injury Epidemiology and Prevention, Columbia University Irving Medical Center, 622 West 168th St, PH5-505, New York, NY 10032 USA; 4 Department of Psychiatry, Columbia University College of Physicians and Surgeons, New York, NY USA; 5 Department of Biostatistics, Columbia University Mailman School of Public Health, New York, NY USA; 6 Bassett Research Institute, Cooperstown, NY USA; 7 University of Michigan Transportation Research Institute, Ann Arbor, MI USA; 8 The Center for Advancing Transportation Leadership and Safety (ATLAS Center), Ann Arbor, MI USA; 9 Department of Emergency Medicine, University of Colorado School of Medicine, Aurora, CO USA; 10 Department of Epidemiology, Colorado School of Public Health, University of Colorado Anschutz Medical Campus, Aurora, CO USA; 11 Department of Health, Behavior, and Society, Johns Hopkins University Bloomberg School of Public Health, Baltimore, MD USA; 12 Department of Family and Preventive Medicine, University of California San Diego, La Jolla, CA USA

**Keywords:** Aging, Beers criteria, Driving safety, Older adults, Potentially inappropriate medications

## Abstract

**Background:**

Potentially Inappropriate Medication (PIM) use has been studied in a variety of older adult populations across the world. We sought to examine the prevalence and correlates of PIM use in older drivers.

**Methods:**

We applied the American Geriatrics Society 2015 Beers Criteria to baseline data collected from the “brown-bag” review of medications for participants of the Longitudinal Research on Aging Drivers (LongROAD) study to examine the prevalence and correlates of PIM use in a geographically diverse, community-dwelling sample of older drivers (*n* = 2949). Proportions of participants who used one or more PIMs according to the American Geriatrics Society 2015 Beers Criteria, and estimated odds ratios (ORs) and 95% confidence intervals (CIs) of PIM use associated with participant characteristics were calculated.

**Results:**

Overall, 18.5% of the older drivers studied used one or more PIM. The most commonly used therapeutic category of PIM was benzodiazepines (accounting for 16.6% of the total PIMs identified), followed by nonbenzodiazepine hypnotics (15.2%), antidepressants (15.2%), and first-generation antihistamines (10.5%). Compared to older drivers on four or fewer medications, the adjusted ORs of PIM use were 2.43 (95% CI 1.68–3.51) for those on 5–7 medications, 4.19 (95% CI 2.95–5.93) for those on 8–11 medications, and 8.01 (95% CI 5.71–11.23) for those on ≥12 medications. Older drivers who were female, white, or living in urban areas were at significantly heightened risk of PIM use.

**Conclusion:**

About one in five older drivers uses PIMs. Commonly used PIMs are medications known to impair driving ability and increase crash risk. Implementation of evidence-based interventions to reduce PIM use in older drivers may confer both health and safety benefits.

**Trial registration:**

Not applicable.

## Background

The Beers Criteria lists potentially inappropriate medications (PIMs) that should *generally* be avoided in older adults because they are ineffective or their risk of adverse effects outweighs the benefit. First developed in 1991 [1], the Beers Criteria has since been revised five times, with the latest version published by the American Geriatrics Society (AGS) in 2019 [2]. Development of the Beers Criteria was based on systematic review of research evidence and expert panel consensus reached through modified Delphi methods, which involved a two-round survey of select experts in geriatrics and pharmacotherapy on various medications and their adverse effects [2]. In addition to the list of PIMs that should generally be avoided in older adults, the Beers Criteria includes medications that should be avoided in older adults with specific diseases or syndromes (e.g., non-steroidal anti-inflammatory drugs [NSAIDs] and COX-2 inhibitors for older adults diagnosed with heart failure because of the risk of fluid retention and exacerbation of cardiac symptoms), and medications that should be used with caution in specific populations (e.g., clopidogrel for adults aged 75 years and older because of the heightened risk of bleeding). The Beers Criteria has also identified drugs for which the dose should be adjusted based on the patient’s renal function or concurrent use of other drugs that may result in drug-drug interactions [2].

Although the Beers Criteria was originally designed as a clinical tool for reducing PIM use and related harms in nursing home residents, it has evolved into an integral part of healthcare policy and best practice in geriatrics [3], and is applicable to all older adults except those in palliative and end-of-life care. Adherence to the Beers Criteria is also used as a measure of healthcare quality by the National Committee for Quality Assurance [3]. In the past two decades, numerous studies have used the Beers Criteria to examine the prevalence of PIM use and associated health consequences in different population groups, such as residents of long-term care facilities, ambulatory care patients, patients filling prescriptions at pharmacies, and community-dwelling older adults [3, 4]. Because the scope of the Beers Criteria has expanded over the years to cover more medications and clinical scenarios, studies based on different versions of the Beers Criteria are not directly comparable. The reported prevalence of PIM use in older adults varies with study settings, ranging from about 20% in community-dwelling older adults [5, 6] to about 50% in older adults presenting to primary care clinics [7], and over 70% in older adult inpatients [8, 9]. Among the most commonly used PIMs are NSAIDs, antihistamines, and benzodiazepines [8, 10]. Older adults in poor health who are taking a large number of medications are at increased risk of PIM use [6, 10]. Use of PIMs in older adults has been recognized as an important cause of adverse drug reactions [11, 12] and excess healthcare costs [13], and has been linked to increased risk of hospitalization and death [4].

Medication use in older drivers is common [14]. According to the 2011 National Health and Aging Trends Study, over 90% of older drivers were taking at least one prescription medication and two-thirds were taking two or more medications [15]. As aging of the US population continues, the effects of medications on driving safety are of increasing concern. Although a variety of medications—such as prescription opioids, antihistamines, antidepressants, benzodiazepines and sleep medications—have been linked to increased crash risk [16–18], little is known about the magnitude of PIMs in the driver population and the implication of PIMs for driving safety. The purpose of the present study is to examine the prevalence and correlates of PIM use in a large sample of community-dwelling older drivers.

## Methods

Data for this study came from the Longitudinal Research on Aging Drivers (LongROAD) project − a multisite prospective cohort study. The LongROAD project was designed to address major questions pertaining to the safety and wellbeing of older drivers, such as the effects of medical conditions and medications on driving behavior and safety, and the determinants and health consequences of driving cessation during the process of aging. Between July 2015 and March 2017, the research team recruited a total of 2990 active drivers aged 65–79 years from primary care clinics or healthcare systems in five study sites (Ann Arbor, MI; Baltimore, MD; Cooperstown, NY; Denver, CO; and San Diego, CA). The local Institutional Review Board at each site approved this study. Following informed consent, each driver was assessed at baseline with standardized research protocols and instruments, including a baseline questionnaire, medical record abstraction, functional tests (e.g., grip strength) and a “brown-bag” review of medications. For the latter, research staff instructed the study participants to bring all current medications (both prescribed and over-the-counter) and supplements with them for review; detailed information was collected about each medication used. The study design and research protocol for the LongROAD project is described in detail elsewhere [19].

Medication data collected at baseline were coded according to the pharmacologic/therapeutic classification system established by the American Society of Health-Systems Pharmacists in the American Hospital Formulary Service (AHFS) Clinical Drug Information [20]. The AHFS classification system groups medications with similar pharmacologic, therapeutic, and chemical characteristics in a four-tier hierarchy, with 31 possible categories in the first tier, 189 in the second tier, 269 in the third tier, and 105 in the fourth tier [20]. Baseline medication data were available for 2949 (98.6%) of the 2990 study participants. A total of 24,690 medications were recorded from the “brown-bag” review at baseline; of them, 22,856 (92.6%) were coded successfully with the AHFS classification system. Non-coded medications included food-like items (e.g., flaxseed oil, protein), homeopathic products (e.g., herbs and spices), and other supplements (e.g., witch-hazel, glucosamine) [21].

The 2015 AGS Beers Criteria for PIM use in older adults (the latest version at the time of this study) was applied to the AHFS-coded medication data to identify PIMs in the study sample [22]. Where necessary, diagnosis data from the study participant’s medical records and self-reported health conditions were reviewed to confirm that the criteria specified in the 2015 AGS Beers Criteria were met. Included in the analysis were PIMs that should generally be avoided, exclusive of proton-pump inhibitors. The 2015 AGS Beers Criteria recommends avoiding long-term (> 8 weeks) use of proton-pump inhibitors except for high-risk patients (e.g., those on oral corticosteroids or chronic NSAIDs). Proton-pump inhibitors, which were used by 21.7% (638 participants) in the study sample, were excluded from the analysis because data from the “brown-bag” review were insufficient to accurately assess the duration of the scheduled use of the medication and users’ risk status. Also excluded from the analysis were: 1) medications for which an accurate assessment of inappropriateness could not be made due to a lack of clinical data on comorbid conditions, indication for use, toxicity, and/or route of administration; 2) medications to avoid for older adults with specific diseases or syndromes; 3) medications to be used with caution due to drug-drug interactions; and 4) anti-infective medications that should be avoided or have their dosage adjusted according to renal function levels [22].

Prevalence of PIM use was calculated according to demographic and health characteristics. For frailty, a 3-category variable was created based on the Fried frailty phenotype: 0, not frail; 1–2, pre-frail; and 3–5, frail [23]. Urbanicity of participant residence was classified using rural-urban commuting area (RUCA) codes derived from ZIP codes in home addresses: urban (RUCA codes 1 and 1.1 [metropolitan core]); suburban (RUCA codes 2, 2.1 and 3 [metropolitan area non-core]); and rural (RUCA codes 4–10 [micropolitan, small towns or rural]) [24].

Differences in the prevalence of PIM use across variables were assessed with chi-square tests. All the covariates of interest and statistical significance at *p* = 0.05 level in the bivariate analysis, except the variable indicating study site, were included in the final multivariable logistic regression model to obtain the adjusted odds ratios (ORs) and their corresponding 95% confidence intervals (CIs) of PIM use.

## Results

Overall, 545 out of the 2949 study participants with medication data available used at least one PIM, yielding a point prevalence of 18.5%. The prevalence of PIM use varied significantly with age, sex, race/ethnicity, and marital status (Table 1). Specifically, higher prevalence of PIM use was found in drivers who were female, white, or not currently married (Table 1). The most pronounced difference in the prevalence of PIM use with regard to demographic characteristics was between sexes, with female drivers being nearly twice as likely as male drivers to use PIMs (23.8% vs. 12.4%, *p* < 0.0001). PIM use was not significantly associated with education or household income levels.

**Table 1:** Prevalence of Potentially Inappropriate Medication (PIM) use in older drivers by demographic characteristics, the Longitudinal Research on Aging Drivers (LongROAD) study

Characteristic	No. of drivers	No. of drivers using one or more PIM^a^	Prevalence (%)	*p*-value
Overall	2949	545	18.5	
Age at baseline (years)				0.244
65–69	1222	225	18.4	
70–74	1027	177	17.2	
75–79	700	143	20.4	
Sex				0.000
Male	1382	172	12.4	
Female	1567	373	23.8	
Race/Ethnicity				0.004
White, non-Hispanic	2526	491	19.4	
Black, non-Hispanic	207	24	11.6	
Hispanic	80	15	18.8	
Asian	65	5	7.7	
Other	71	10	14.1	
Marital Status				0.003
Married	1850	307	16.6	
Divorced	439	105	23.9	
Widowed	371	81	21.8	
Never married	128	23	18.0	
Other	161	29	18.0	
Education				0.248
Less than high school	70	13	18.6	
High school	265	43	16.2	
Some college/Associate’s degree	715	152	21.3	
Bachelor’s degree	691	126	18.2	
Advanced degree	1208	211	17.5	
Household Income in the previous year				0.332
< $20,000	131	31	23.7	
$20,000–$49,999	633	118	18.6	
$50,000–$79,999	710	133	18.7	
$80,000–$99,999	427	67	15.7	
≥ 100,000	942	174	18.5	
Frailty score				0.286
Not frail	1205	215	17.8	
Pre-frail	1636	302	18.5	
Frail	85	21	24.7	
Study Site				0.005
Ann Arbor, MI	595	102	17.1	
Baltimore, MD	583	100	17.2	
Cooperstown, NY	595	88	14.8	
Denver, CO	577	124	21.5	
San Diego, CA	599	131	21.9	

^a^Includes 450 drivers using one PIM, 76 using two PIMs, 15 using three PIMs, 3 using four PIMs, and 1 using five PIMs

When measured by frailty score, 41.2% of the study participants was classified as not frail, 55.9% as pre-frail, and 2.9% as frail. The prevalence of PIM use was 17.8% for those in the “not frail” group, 18.5% in the “pre-fail” group and 24.7% in the “frail” group (*p* = 0.286; Table 1).

**Fig. 1:**
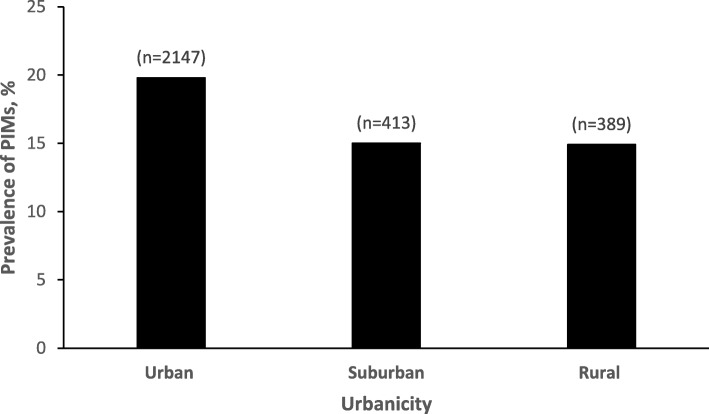
Prevalence of Potentially Inappropriate Medication (PIM) use by urbanicity in older adult drivers, the Longitudinal Research on Aging Drivers (LongROAD) study

The prevalence of PIM use increased progressively with the total number of medications used. Use of PIMs was highest among older drivers taking 12 or more medications; over one-third (34.3%) of this group used PIMs, compared with 21.4% of those on 8–11 medications, 13.4% of those on 5–7 medications and 6.0% of those on four or fewer medications (*p* < 0.001; Fig. 1).

**Fig. 2:**
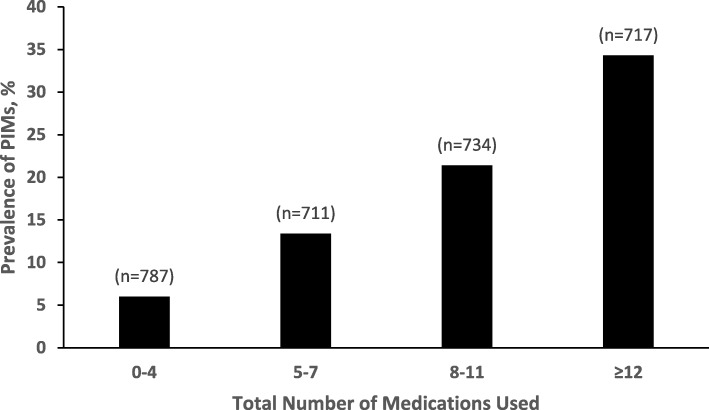
Prevalence of Potentially Inappropriate Medication (PIM) use by total number of medications used in older adult drivers, the Longitudinal Research on Aging Drivers (LongROAD) study

The prevalence of PIM use varied with study sites, ranging from 14.8% for drivers recruited in Cooperstown, NY to 21.9% for drivers recruited in San Diego, CA (Table 1). Almost three-quarters (72.8%) of the drivers studied were living in urban areas, 14.0% in suburban areas, and 13.2% in rural areas. The prevalence of PIM use among older drivers in urban areas was 20.1%, significantly higher than in suburban areas (13.6%) and rural areas (14.7%) (*p* = 0.001; Fig. 2).

Use of multiple PIMs was fairly common. Of the 545 PIM users, 95 (17.4%) used two or more PIMs. The most frequently used therapeutic category of PIM was short- and intermediate acting benzodiazepines such as alprazolam, lorazepam and temazepam, accounting for 16.6% of the total PIMs identified. Other frequently used PIMs were nonbenzodiazepine hypnotics (e.g., eszopiclone, zolpidem and zalepon; 16.4%), antidepressants (e.g., amitriptyline and clomipramine; 15.2%), first-generation antihistamines (e.g., chlorpheniramine and diphenhydramine; 10.5%), estrogens (oral and topical; 10.4%), skeletal muscle relaxants (e.g., carisoprodol and metaxalone; 8.6%), and NSAIDs (e.g., ibuprofen and naproxen; 7.4%) (Table 2). Together, these seven therapeutic categories accounted for 85.1% of the total PIMs.

**Table 2:** Sex-specific frequencies of potentially inappropriate medications by therapeutic category in older drivers, the Longitudinal Research on Aging Drivers (LongROAD) study

Therapeutic Category	Males No. (%)	Females No. (%)	Total^a^ No. (%)
Benzodiazepines	27 (14.0)	83 (17.6)	110 (16.6)
Nonbenzodiazepine Hypnotics	45 (23.3)	64 (13.6)	109 (16.4)
Antidepressants	24 (12.4)	77 (16.3)	101 (15.2)
First-Generation Antihistamines	22 (11.4)	48 (10.2)	70 (10.5)
Estrogens (Oral and Patch)	0 (0.0)	69 (14.7)	69 (10.4)
Skeletal Muscle Relaxants	15 (7.8)	42 (8.9)	57 (8.6)
NSAIDs (Oral)	22 (11.4)	27 (5.7)	49 (7.4)
Antispasmodics	9 (4.6)	24 (5.1)	33 (5.0)
Antipsychotics	4 (2.1)	12 (2.6)	16 (2.4)
Sulfonylureas (Long duration)	8 (4.1)	3 (0.6)	11 (1.6)
Other^b^	17 (8.8)	22 (4.7)	39 (5.9)
Total	193 (100.0)	471 (100.0)	664 (100.0)

^a^Includes 450 drivers using one PIM, 76 using two PIMs, 15 using three PIMs, 3 using four PIMs, and 1 using five PIMs

^b^Includes 9 drivers (0 M, 9 F) on barbiturates, 6 (5 M, 1 F) on dronedarone, 6 (5 M, 1 F) on insulin (sliding scale), 4 (0 M, 4 F) on nitrofuratonin, 3 on androgens (3 M, 0 F), 3 (1 M, 2 F) on metoclopramide, 3 (2 M, 1 F) on desmopressin, 2 (0 M, 2 F) on peripheral alpha-1 blockers, 2 (1 M, 1 F) on mineral oil (oral), and 1 (0 M, 1 F) on antiparkinsonian agents

Multivariable logistic regression modeling revealed that the total number of medications used was most strongly associated with PIM use; relative to older drivers on four or fewer medications, the adjusted ORs of PIM use were 2.43 (95% CI 1.68–3.51) for those on 5–7 medications, 4.19 (95% CI 2.95–5.93) for those on 8–11 medications, and 8.01 (95% CI 5.71–11.23) for those on ≥12 medications. When the total number of medications used was treated as a continuous variable in the multivariable logistic regression model, the odds of PIM use increased 13% (adjusted OR 1.13; 95% CI 1.11–1.15) with each unit increase in the total number of medications. Other variables significantly associated with PIM use were sex, race/ethnicity, and urbanicity. Specifically, older drivers who were female, white, or living in urban areas were at significantly increased risk of PIM use (Table 3). There were no significant interaction effects on PIM use between these variables. Because older drivers representing rural areas in the study sample came primarily from Cooperstown, NY, study site was not included in the multivariate analysis.

**Table 3:** Adjusted Odds Ratios (ORs) and 95% Confidence Intervals (CIs) of Potentially Inappropriate Medication (PIM) use in older drivers by risk markers, the Longitudinal Research on Aging Drivers (LongROAD) study

Risk Marker	Adjusted OR^a^	95% CI
Sex		
Male	1.00	reference
Female	2.05	1.65–2.55
Race/Ethnicity		
White, non-Hispanic	1.00	reference
Black, non-Hispanic	0.42	0.26–0.66
Hispanic	0.86	0.47–1.58
Asian	0.36	0.14–0.92
Other	0.61	0.30–1.22
Total number of medications used		
0–4	1.00	reference
5–7	2.43	1.68–3.51
8–11	4.19	2.95–5.93
≥ 12	8.01	5.71–11.23
Urbanicity		
Rural	1.00	reference
Suburban	0.90	0.60–1.37
Urban	1.61	1.17–2.21

*OR* Odds Ratio; 95% CI = 95% Confidence Interval

^a^Adjusted for age, marital status and frailty phenotype in addition to variables in the table

## Discussion

The prevalence of PIM use found in this study, 18.5%, is lower than reported in studies conducted in clinical settings involving different patient groups [7, 8] but is generally comparable to findings from studies in community-dwelling older adults and non-institutionalized Medicare beneficiaries [13, 25]. The relatively low use of PIMs reported in this study is likely due to three factors. First, the study sample was comprised of older drivers who were more active and healthier than the general older adult population [19]. For example, less than 3% of the drivers included in this study were classified as frail according to the Fried frailty phenotype, compared with about 11% in the community dwelling older adult population [26]. Second, the prevalence of PIM use reported in this study was based on all participants with medication review data, including those who were found to be not taking any medications (3.3% of the study sample). Previous studies of PIMs were mostly restricted to older adults who were taking one or more medications. Finally, the estimated prevalence of PIM use in this study is likely conservative because we excluded proton-pump inhibitors and a few other medications on the list of PIMs due to insufficient clinical data necessary for applying the AGS 2015 Beers Criteria to these medications, such as duration, dosage, and user’s risk status. Several studies have reported that proton-pump inhibitors are one of the most commonly used PIMs in older adults [9, 25]. Our data indicate that 21.7% of the older drivers included in this study were taking proton-pump inhibitors, although it is unclear whether this therapeutic category of PIM was used inappropriately according to the AGS 2015 Beers Criteria.

Previous studies have identified several demographic and clinical characteristics associated with PIM use, including age, sex, race, health status, and total number of medications used. Findings from our study are generally consistent with the existent research literature. Because of the favorable health status of study participants, associations of age and health status (as measured by frailty status) with PIM use did not reach statistical significance. As the participants grow older and more frail, we expect that the associations of advancing age and declining health status with PIM use may manifest in the follow-up data. Although other researchers have reported a higher prevalence of PIM use in older women than in older men [9, 25, 27], our study reveals that the excess prevalence of PIMs in women was due in a large part to use of estrogens. Furthermore, data from the multisite LongROAD project allowed us to identify the geographical gradient in PIM use. Specifically, we found that older drivers living in urban areas were about 60% more likely to use PIMs than those living in rural and suburban areas, with adjustment for demographic characteristics, frailty status, and total number of medications used. Prior studies of urbanicity and PIM use by older adults have produced inconsistent results. Inappropriate medication prescribing has sometimes been reported as similar in urban and rural locations [6, 28] or as more common for urban patients [29] or rural patients [30]. An analysis of Veterans Administration data on elder veterans found that urban-rural differences in prescribing quality varied by region of the country, with inappropriate prescribing associated with rural locations in the South and Northeast but urban locations in the West [31]. Previous research suggests that older adults in the West, Midwest, and South regions are at greater risk of using PIMs than in the Northeast [32]. Our finding of urbanicity as a risk marker, if confirmed by other researchers, may help enhance intervention programs by better targeting populations at high-risk for PIM use to improve health outcomes in older adults.

The PIMs included in the Beers Criteria should generally be avoided in older adults because they are therapeutically ineffective or pose an exceptionally high risk of adverse effects, such as delirium, internal bleeding, and injury resulting from falls. For some PIMs, safer alternative medications or non-pharmaceutical therapies are available. Previous studies examining the consequences of PIM use were focused on adverse health outcomes, such as drug reactions, complications, morbidity and mortality [4, 33]. Our study provides, for the first time, detailed data on PIMs used by older drivers. Most of the commonly used PIMs identified in this study, such as benzodiazepines, nonbenzodiazepine hypnotics, antidepressants, and first-generation antihistamines, are medications known to impair driving performance and increase crash risk [17, 18, 34, 35]. For example, meta-analyses have shown that use of benzodiazepines is associated with 60–80% increased risk of crash involvement and 40% increased risk of crash culpability and that older adults are particularly susceptible to the deleterious effect of antidepressants on driving safety [18, 34]. Our study indicates that about 11% of older drivers use benzodiazepines, nonbenzodiazepine hypnotics, antidepressants, or first-generation antihistamines – PIMs that can impair driving ability and increase crash risk.

There are several limitations with this study. First, the Beers Criteria is a clinical tool designed to identify and evaluate *potentially* inappropriate medications that should *generally* be avoided in older adults. These qualifiers are necessary because there could be particular clinical circumstances under which use of a PIM might be justified. Second, the prevalence of PIMs reported in this study is likely a conservative estimate because our analysis was restricted to PIMs that should generally be avoided in older adults and because our analysis excluded proton-pump inhibitors due to insufficient data about the duration of usage and the user’s risk status. Other studies reporting much higher prevalence of PIM use may have included additional medications identified in the AGS 2015 Beers Criteria for their risks of drug-disease and drug-drug interactions, such as opioids for older adults with a history of falls or fractures [36, 37]. Finally, this study relied on an analysis of medication review data collected as part of the baseline assessment in the LongROAD project and therefore is cross-sectional in nature. Future research should incorporate prospectively collected follow-up data to understand the trajectory of PIM use during the process of aging and the relationships between PIM use and driving outcomes, such as safety behavior and crash risk.

## Conclusion

In summary, this study provides valuable empirical evidence for understanding the magnitude of PIM use in older drivers and sheds light on the factors associated with PIM use and specific medications involved. Our conservative estimate indicates that nearly a fifth of older drivers use one or more PIMs. The odds of PIM use increases with the total number of medications being taken and is particularly high among white, female older drivers living in urban areas. The PIMs most commonly used by older drivers are benzodiazepines, nonbenzodiazepine hypnotics, antidepressants, and first-generation antihistamines, all of which have been linked to driving impairment and increased crash risk. Implementation of evidence-based interventions, such as computer-based alerts and prescription rule restrictions [38], may reduce PIM use and improve health outcomes and driving safety in older adults.

## Data Availability

Data used in this study are available from the authors upon reasonable request and with permission of the AAA Foundation for Traffic Safety.

## Abbreviations

AGS: American Geriatrics Society
AHFS: American Hospital Formulary Service
CI: Confidence interval
LongROAD: Longitudinal Research on Aging Drivers
NSAID: Non-steroidal anti-inflammatory drug
OR: Odds ratio
PIM: Potentially inappropriate medication
RUCA: Rural-urban commuting area
